# If you thought you were good with a scalpel, you should try this

**DOI:** 10.1186/1757-1146-4-S1-P39

**Published:** 2011-05-20

**Authors:** Kate McCabe, Alicia Ball

**Affiliations:** 1 Peninsula Health, Frankston, Vic, 3199, Australia

## 

In 2010 Peninsula Health was one of four Victorian health services provided with a Department of Health New Technology Grant worth $100, 000. The grant funded the implementation and evaluation of the use of Low Frequency Ultrasonic Debridement (LFUD) in wound healing. The LFUD device, Sonoca, has been developed for use on diabetic foot ulcers, pressure ulcers, venous leg ulcers and dehisced wounds. A report by the Department of Health and Aging (2007) acknowledged that current evidence indicates that the use of LFUD promotes significantly enhanced wound healing compared to using conventional wound dressing with antiseptic agents. It will be some time before we have the data available to evaluate healing rates with the use of LFUD. In the interim, we have been able to impress all parties involved in wound management with the improved quality of our debridement and wound bed preparation. LFUD provides all the benefits of conventional debridement in addition to: returning the wound to the acute phase by vasodilation; stimulating fibroblasts, macrophages and endothelial cells; and breaking down biofilm. There is also evidence of LFUD promoting faster wound healing, reducing pain on debridement and having antimicrobial effects. The use of LFUD is time-consuming and expensive compared with conservative sharps debridement. However, LFUD is quick and inexpensive compared with theatre debridement or amputation. I believe this technology will be an indispensible part of the podiatrist’s tool kit in years to come.

